# Repurposing dyphylline as a pan-coronavirus antiviral therapy

**DOI:** 10.4155/fmc-2021-0311

**Published:** 2022-04-07

**Authors:** Yining Wang, Sajjan Rajpoot, Pengfei Li, Marla Lavrijsen, Zhongren Ma, Nik Hirani, Uzma Saqib, Qiuwei Pan, Mirza S Baig

**Affiliations:** ^1^Department of Gastroenterology & Hepatology, Erasmus MC-University Medical Center, Rotterdam, 3015 CN, The Netherlands; ^2^Department of Biosciences & Biomedical Engineering (BSBE), Indian Institute of Technology Indore (IITI), Simrol, Indore, 453552, India; ^3^Biomedical Research Center, Northwest Minzu University, Lanzhou, 730030, China; ^4^MRC Centre for Inflammation Research, Queen's Medical Research Institute, University of Edinburgh, Edinburgh, EH164TJ, UK; ^5^Department of Chemistry, Indian Institute of Technology Indore (IITI), Simrol, Indore, 453552, India

**Keywords:** drug discovery, dyphylline, human coronaviruses (HCoVs), Mpro, repurposing

## Abstract

**Background:** In the last two decades, the world has witnessed the emergence of zoonotic corona viruses (CoVs), which cause mild to severe respiratory diseases in humans. Human coronaviruses (HCoVs), mainly from the alpha-CoV and beta-CoV genera, have evolved to be highly pathogenic, such as SARS-CoV-2 causing the COVID-19 pandemic. These coronaviruses carry functional enzymes necessary for the virus life cycle, which represent attractive antiviral targets. **Methods & Results:** We aimed to therapeutically target the main protease (Mpro) of HCoV-NL63 and HCoV-229E (from alpha-CoV genus) and HCoV-OC43 and SARS-CoV-2 (from beta-CoV genus). Through virtual screening, we identified an FDA-approved drug dyphylline, a xanthine derivate, that binds to the catalytic dyad residues; histidine and cystine of the Mpro structures. Importantly, dyphylline dose-dependently inhibited the viral replication in cell culture models infected with the viruses. **Conclusion:** Our findings support the repurposing of dyphylline as a pan-coronavirus antiviral agent.

Coronaviruses (CoVs) are a group of large, enveloped, non-segmented, single-stranded, positive-sense RNA viruses, which carry the largest genome among all known RNA viruses, typically ranging from 27 kb to 32 kb [1,2]. As per the International Committee for Taxonomy of Viruses, CoVs are classified under four genera, namely *alpha coronavirus*, *beta coronavirus*, *gamma coronavirus*, and a novel *delta coronavirus* (Figure 1A). Broadly, the alpha- and beta-CoV infects mammals, while gamma-CoV infects avian species and delta-CoV infects both mammals and avian species [2–4]. Human coronaviruses (HCoVs), which originate from animals, mainly belong to alpha- and beta-CoV. Currently, there are seven known HCoVs; two alpha-CoVs, namely HCoV-229E and HCoV-NL63 and five beta-CoVs namely HCoV-OC43, HCoV-HKU1, MERS-CoV, SARS-CoV, and SARS-CoV-2 [5,6]. They cause mild to severe respiratory illness, such as SARS-CoV-2 causing the ongoing COVID-19 pandemic.

**Figure 1.**
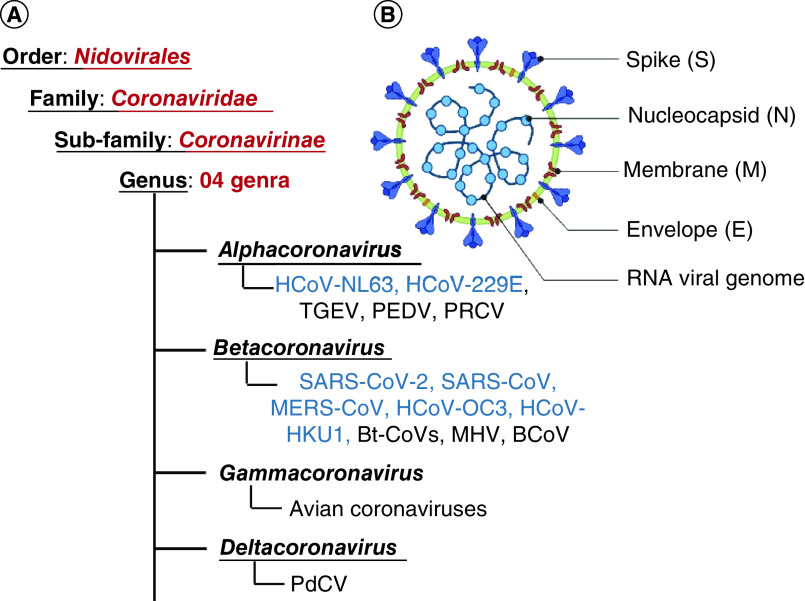
Classification of coronaviruses. **(A)** Coronavirus classification showing its four genera. **(B)** Virion organization representing the structural proteins. The human Coronaviruses (HCoVs) are highlighted in blue. HCoV (Human coronavirus), HCoV-NL63 (Netherlands 63), HCoV-229E (named after specimen code 229E) TGEV (Porcine transmissible gastroenteritis coronavirus), PEDV (Porcine epidemic diarrhea virus), PRCV (Porcine respiratory coronavirus), SARS-CoV (Severe acute respiratory syndrome coronavirus), MERS-CoV (Middle East respiratory syndrome coronavirus), HCoV-OC43 (Organ culture 43), HCoV-HKU1 (Hong Kong University 1), Bt-CoVs (Bats coronavirus), MHV (Mouse hepatitis coronavirus), BCoV (Bovine coronavirus), and PdCV (Porcine deltacoronavirus).

Besides the launch of several effective vaccines against SARS-CoV-2, treatment mainly includes the repurposing of existing antimalarial, antiviral, antibiotic, immunosuppressant and monoclonal antibodies agents such as chloroquine, hydroxychloroquine, lopinavir, ritonavir, remdesivir, ribavirin, favipiravir, molnupiravir, azithromycin, teicoplanin, cyclosporine and tocilizumab, many of them have entered in clinical trials [7–10]. However, only two oral antiviral drugs molnupiravir (from Merck) and ritonavir boosted nirmatrelvir (known as paxlovid from Pfizer) have been recently given the emergency use authorization (EUA) by United States Food and Drug Administration (US FDA) for COVID-19 whereas others are underdevelopment [11]. Their uses are limited in patients based on situation where other FDA authorized treatments are inaccessible while both drugs are not authorized to be taken consecutively for more than 5 days. Besides, paxlovid has significant and complex drug–drug interaction potential with concomitant medication due to the presence of ritonavir, which may lead to drug toxicity, whereas other concerns are raised against molnupiravir as described [11].

Besides the structural proteins (Figure 1B), coronaviruses also encode several non-structural proteins (nsps) including RNA-dependent RNA polymerase (RdRp), main protease ([Mpro] also known as 3-chymotrypsin-like protease [3-CLpro] or nsp5), and papain-like protease (PLpro) (or nsp3) crucial for translation, protein processing and replication process of the virus [5,12] and these nsps have been highly coveted as therapeutic targets to counter illness caused by coronaviruses [13–19]. Mpro is largely conserved among HCOVs and has been widely explored for developing anti-coronavirus agents. The catalytic dyad of histidine and cystine in the catalytic domain of Mpro plays a crucial role in protease function [20–25]. Therefore, identifying molecules that can block the catalytic dyad of Mpro holds promising therapeutic potential.

In this study, we aimed to identify Mpro inhibitors from existing approved medications through virtual screening and experimental validation in cell culture models infected with the virus. We employed four HCoVs including HCoV-229E, HCoV-NL63, HCoV-OC43 and SARS-CoV-2, for identifying Mpro inhibitors with pan-coronavirus antiviral activity. We showed that the virtually screened dyphylline, a xanthine derivative, is able to block the catalytic dyad of all the four Mpro structures and our *in vitro* results demonstrated the promising anti-coronaviral property of the dyphylline for the first time. The therapeutic potential of xanthine derivatives has long since been known [26,27]. Xanthine are naturally occurring compounds found in plant products such as coffee, cocoa beans, and tea. While xanthine derivatives are a group of alkaloids which resemble the naturally occurring xanthine such as caffeine, theobromine and methylxanthine, mainly act as bronchodilators by relaxing the smooth muscles [28–30]. Xanthine derivatives may also act as an anti-inflammatory agents regulating cytokines release or activation of histone deacetylase. These drugs very rarely cause drug-induced liver injury or other side effects besides minor side effects such as headache, dizziness, etc and are used for treatment of asthma and chronic obstructive lung disease [30]. Dyphylline pharmacologic action is similar to theophylline and other derivatives of this group and acts as bronchodilator [31,32]. Therefore, the existing use of dyphylline as bronchodilator in respiratory disease may have the synergistic effect in HCoV infected patients showing related symptoms. It suggests that further study would be interesting in order to underscore its therapeutic efficacy in animal models and for clinical studies.

## Materials & methods

### Structure retrieval, homology-based 3D structure modeling & its preparation

A molecular docking study was performed between FDA-approved drugs and Mpro of four coronavirus (CoVs) strains. The structure of Mpro of three coronavirus strains SARS-CoV-2, HCoV-NL63, and HCoV-229E with PDB Id 6M2Q, 5GWY and 2ZU2 were retrieved from the PDB database (www.rcsb.org/). For HCoV-OC43 Mpro structure, homology-based 3D structure modeling was performed in MODELLER 10.1 [33]. For template search, HCOV-OC43 Mpro sequence was obtained from Uniprot Id P0C6X6 (www.uniprot.org/blast/?about=P0C6X6[3247-3549]&key=Chain&id=PRO_0000283830). The HCoV-HKU1 Mpro structure (PDB Id 3D23, Chain A) was selected as a template based on the highest sequence identity (82.33% identity with 99% of query sequence cover) in NCBI protein blast (https://blast.ncbi.nlm.nih.gov/Blast.cgi) using protein data bank proteins as a database. The query and template sequence was aligned in the CLUSTALW tool (www.genome.jp/tools-bin/clustalw) in protein information resource (PIR) format for structure modeling. A structure with the best discrete optimized protein energy (DOPE) score was subjected for loop refining in Modeler 10.1 [33] and its stereochemical analysis was performed in PROCHECK [34] and ProtSAV [35] for the Ramachandran plot and overall quality assessment of the structure, respectively. The RMSD value between template and model structure was computed in the Chimera tool [36]. Finally, all four Mpro structures were prepared for docking in Discovery Studio Visualizer [37]. In retrieved structures, the water molecules, heteroatoms and any co-crystallized ligand groups were deleted followed by the addition of polar hydrogen atoms in all structures including modeled Mpro of HCoV-OC43.

### Molecular docking & virtual screening study

The prepared structures of all four Mpro were individually used for the screening of potential drug candidates which can block its catalytic function. The FDA-approved drug library (a total of 1576 drug candidates) was retrieved from the ZINC15 database (https://zinc15.docking.org/) and prepared in OpenBabel tool [38] for docking in AutoDock Vina [39] and SwissDock [40]. The molecular docking was performed in an unbiased blind dock mode, allowing the compounds to find and fit best in the target structure. Further, the catalytic dyad in Mpro of the studied coronavirus strains was considered for screening of the drug candidates and drug binding specifically to all the catalytic residues (H41 and C145 in SARS-CoV-2 and HCoV-OC43 Mpro, and H41 and C144 in HCoV-NL63 and HCoV-229E Mpro, respectively) were selected for analysis. The interaction analysis between drug and protein was performed in Discovery studio visualizer [37].

### Reagents & antibodies

Dyphylline (Bio-Connect, The Netherlands) and molnupiravir (MedChem Express, NJ, USA) were dissolved in phosphate-buffered saline (PBS) and dimethyl sulfoxide (DMSO, Sigma, Zwijndrecht, The Netherlands) respectively. Anti-double-stranded-RNA (dsRNA) antibody (SCIONS J2 monoclonal antibody) was received from English & Scientific Consulting Kft. Anti-mouse IgG (H&l Alexa Fluor^®^594, Abcam) was used as the secondary antibody for the western blot experiments.

### Viruses & cell lines

Monkey LLCMK-2 cells were cultured in minimal essential medium containing Earle's salt (MEM; Gibco, NY, USA) with 8% (vol/vol) heat-inactivated fetal calf serum (FCS, Sigma-Aldrich, MO, USA), 1% (vol/vol) non-essential amino acids (Sciencell, CA, USA), 0.1% (vol/vol) L-Glutamine (Lonza, Verviers, Belgium), 100 IU/ml penicillin and 100 mg/ml streptomycin (Gibco). All cell lines used in the study including the Human colon cancer, Caco-2, human hepatoma Huh7, kidney cells from monkeys, Vero-E6 and human adenocarcinomic alveolar basal epithelial cell, A549 were cultured in Dulbecco's modified Eagle medium (DMEM) (Lonza Biowhittaker, Verviers, Belgium) supplemented with 10% (vol/vol) heat-inactivated fetal calf serum (FCS, Sigma-Aldrich), 100 IU/ml penicillin and 100 mg/ml streptomycin (Gibco). Human alveolar cancer cell line Calu-3 were cultured in advanced DMEM/F12 supplemented with 1% (vol/vol) GlutaMAXTM Supplement (Gibco), 10 mM HEPES. The HCoV-NL63 stock was produced by consecutively inoculating the virus onto LLCMK-2 cells. Seasonal coronavirus HCoV-OC43 and HCoV-229E were bought from ATCC (USA) and amplified in Huh7 cells. The SARS-CoV-2 stock was produced as described previously. Cell lines were analyzed by genotyping and confirmed to be mycoplasma negative.

### Virus production & inoculation re-infection assay

LLCMK-2 cells harboring the infectious HCoV-NL63 were seeded into multi-well plates, culturing at 33°C, with 5% CO_2_ for 5–7 days, over 50% of cells have cytopathic effect (CPE), the HCoV-NL63 particles were harvested by repeated freezing and thawing three times and filtered with 0.45 μm filters. Huh7 cells harboring the infectious HCoV-OC43 or HCoV-229E were seeded into multi-well plates, culturing at 33°C, with 5% CO_2_ for 4–6 days. When over 50% of cells have the cytopathic effect (CPE), OC43 or 229E particles were collected by freezing and thawing at least three times. They were then filtered using filters of 0.45 μm diameter. The SARS-CoV-2 stock was made available by the generous efforts of Dr. Bart Haagmans (Department of Viroscience, Erasmus MC). In short, Vero-E6 cells harboring the infectious SARS-CoV-2 were seeded into multi-well plates and incubating the cells at 37°C, with 5% CO_2_ for about 72 h. The culture supernatant was centrifuged, separated and stored in mini aliquots at -80°C temperature. Finally, the cells were seeded into multi-well plates. When the cell confluence was approximately 80%, the culture medium was discarded and the cells were washed thrice with 1 × PBS. HCoV-NL63 virus was harvested and incubated with cells overnight. HCoV-OC43 or HCoV-229E viruses were added, incubated with cells for 2 h at 33°C. SARS-CoV-2 viruses were added, incubated with cells for 1 h at 37°C, with 5% CO_2_. Later, the cells were washed three times with 1 × PBS to remove viruses that remained unattached, followed by incubating them in a culture medium for 48 h. The viral titers were analyzed by TCID50 assay.

### Antiviral drug treatment

LLCMK-2, Huh7 and Caco-2 cells were inoculated with HCoV-NL63 at 0.1 MOI (multiplicity of infection) and later incubated overnight at 33°C. On the other hand, A549 cells were inoculated with HCoV-229E or HCoV-OC43 with similar conditions described above for 2 h. Contrary to this, the Calu-3 cells were inoculated with SARS-CoV-2 at the same MOI described previously and incubated at 37°C for 1 h. The cells were gently washed with PBS a few times in order to remove unattached virus particles followed by treatment with dyphylline for the specified time period. The cells, total RNA or supernatant were collected for further experimentation.

### RNA isolation, cDNA synthesis & qRT-PCR

The total RNA was isolated and quantified using Macherey-Nagel NucleoSpin^®^ RNA II kit (Bioke, Leiden, The Netherlands) and Nanodrop ND-1000 (Wilmington, DE, USA) respectively. cDNA synthesis kit (TaKaRa Bio, Inc., Shiga, Japan) was used to synthesized cDNA. Real-time PCR reactions utilized the SYBR-Green-based real-time PCR (Applied Biosystems^®^, TX, USA) analysis on a StepOnePlusTM System (Thermo Fisher Scientific LifeSciences) platform. The glyceraldehyde 3-phosphate dehydrogenase (GAPDH) gene was used as the reference or the housekeeping gene by normalizing the relative gene expression of the target gene to the expression of the former. The following formula was used:

2^-ΔΔCT^, ΔΔCT = ΔCT sample – ΔCTcontrol (ΔCT = CT [target gene] – CT[GAPDH]).

Controls including the template and reverse transcriptase were included in all qRT-PCR experiments. The primers are listed in Supplementary Table 1.

### MTT assay

The cell lines including the LLCMK-2, Huh7, Caco-2, A549 and Calu-3 were seeded into 96-well tissue culture plates at a frequency of about 1 × 10^4^ cells/well. They were then treated with the specified compounds for 48 h. Cells were incubated with 10 μl 3-(4,5-dimethyl-2-thiazolyl) -2,5-diphenyl-2H-tetrazolium bromide (MTT) at a concentration of 5 mg/ml for 3 h, followed by replacing them with 100 μl DMSO medium for 30 minutes at 37°C. The absorbance was recorded at 490 nm using a microplate absorbance reader (Bio-Rad, CA, USA).

### TCID50 assay

The supernatant carrying the viruses in the cultured cells was harvested by repeated freezing and thawing three times. 50% tissue culture infectious dose (TCID50) assay was used to quantify HCoV-NL63 titer. HCoV-NL63 dilutions of about tenfold were inoculated into the 96-well tissue culture plate of LLCMK-2 cells with 2,000 cells/well. The plate was incubated at 33°C for 5–7 days, followed by examining them for their cytopathic effect (CPE) using a light microscope. Reed-Muench method was used to calculate the TCID50 value.

### Quantification of HCoV-NL63 genome copy numbers

The pCR2.1-TOPO vector (Invitrogen, CA, USA) was used to clone the amplicon of HCoV-NL63 (a fragment of N protein). This led to the generation of a template for the quantification of HCoV-NL63 genome copy numbers. Quick Plasmid Miniprep Kit (Invitrogen, Lohne, Germany) was used to extract the plasmid. Multiple dilutions ranging from 10^-1^ to 10^-8^ were made and later amplified and quantified by qRT-PCR for the generation of a standard curve. The latter was plotted using the log copy number versus the cycle threshold (CT) value (Supplementary Figure 3). The copy numbers were calculated by using the following equation:

$$\text{Copy number}(\text{molecules}/\mu \text{l})=\frac{\text{concentration}(\text{ng}/\mathrm{\mu l})\times 6.022\times 10^{23}(\text{molecules}/\text{mol})}{\text{length of amplicon}\times 640(\text{g}=/\text{mol})\times 10^{9}(\text{ng}/\text{g})}$$

### Confocal fluorescence microscopy

A549 cells cultured were inoculated with HCoV-229E and HCoV-OC43 respectively in an 8-well chamber (cat. no. 80826; ibidi GmbH) at 0.1 MOI, followed by their incubation at 33°C for 2 h. The culture medium was further replaced with another medium containing varied concentrations of dyphylline. The cells were cultured for another 48 h and later fixed with 4% paraformaldehyde in PBS for 10 min. In order to permeabilize them, Triton X-100 at a ratio of 0.2% (vol/vol) was added to them for 10 min. This was further blocked with milk-tween-glycine medium (0.05% tween, 0.5% skim milk and 0.15% glycine) for 1 h, after which they were treated with anti-dsRNA antibody (1:200) diluted in blocking solution at 4°C overnight. Anti-mouse IgG secondary antibodies in 1:1000 dilutions were incubated with the cells for 1 h. Nuclei were stained using the dye, DAPI (4, 6-diamidino-2-phenylindole; Invitrogen) and later the images were taken using the Leica SP5 cell imaging system.

### Statistics analysis

The results are reported as mean ± SEM. The Mann–Whitney test (GraphPad Prism 5; GraphPad Software Inc., La Jolla, CA) was used to calculate the statistical significance of differences between the means. The threshold for statistical significance has been predefined at p ≤ 0.05.

## Results

### Virtual screening of FDA-approved drugs against Mpro of SARS-CoV-2, HCoV-NL63, HCoV-229E & HCoV-OC43 by molecular docking

**Figure 2.**
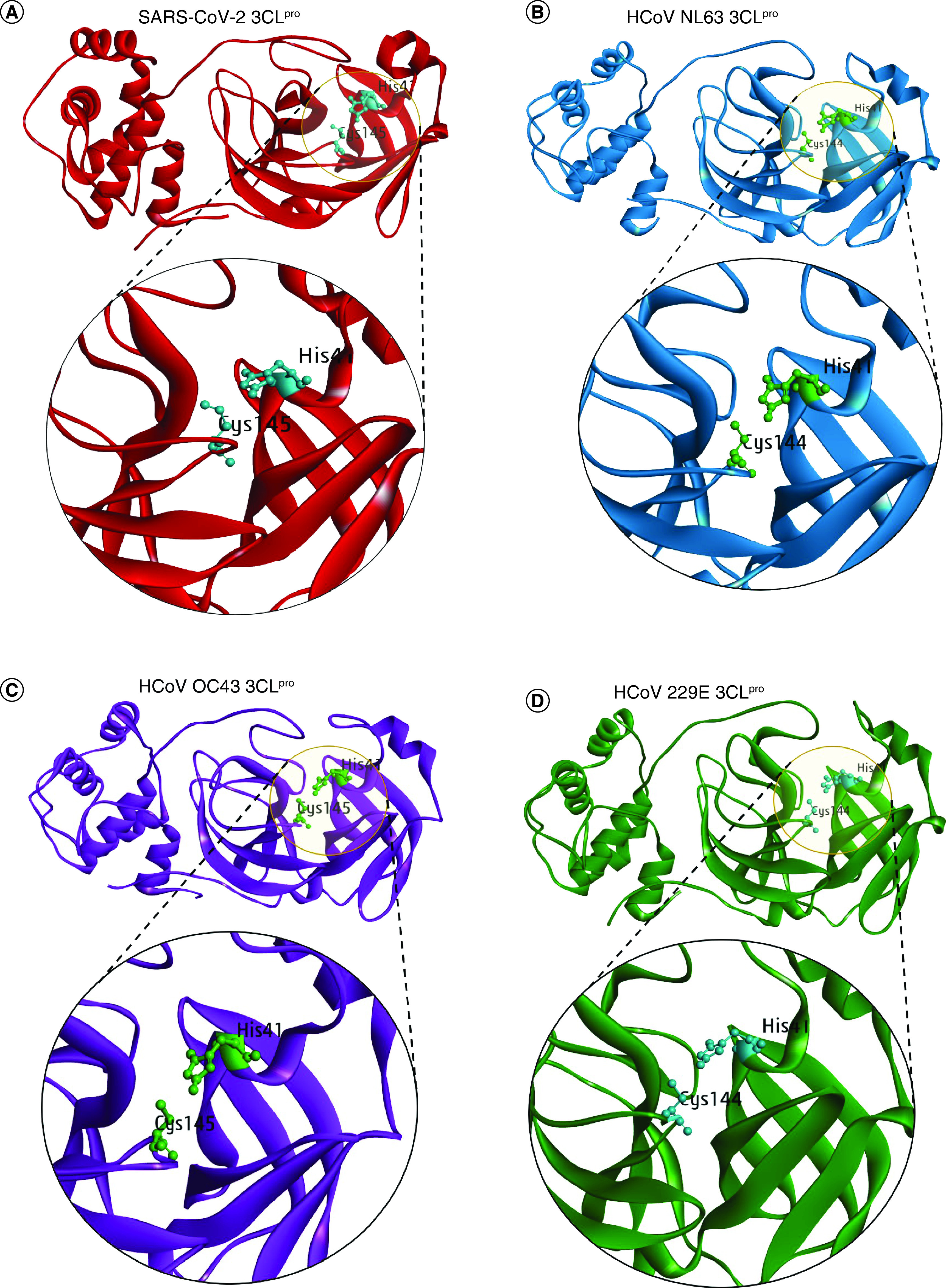
Structural representation of Mpro/3CLpro and its catalytic dyad. **(A)** SARS-CoV-2. **(B)** HCoV-NL63. **(C)** HCoV-OC43. **(D)** HCoV-229E. The Mpro of SARS-CoV-2, HCoV-NL63, and HCoV-229E was obtained from the PDB database with PDB Id 6M2Q, 5GWY, and 2ZU2 while the Mpro structure of HCoV-OC43 was modeled using HCoV-HKU1 as the template in MODELLER 10.1 tool.

For identifying pan-coronavirus antiviral agents, we targeted the Mpro of four HCoVs (SARS-CoV-2, HCoV-NL63, HCoV-OC43, and HCoV-229E) (Figure 2A–D). The Mpro in all four strains contains a catalytic dyad consisting of histidine and cystine residue in its catalytic domain which is crucial for its function [48]. Histidine and Cystine are at the 41st and 145th positions in SARS-CoV-2 [49] and HCoV-OC43 Mpro [50] while at 41st and 144th in HCoV NL-63 [28] and HCoV-229E Mpro [51], respectively (Figure 2).

Therefore, we attempted to screen molecules that can effectively bind to the catalytic dyad to block coronavirus Mpro function. A structure-based molecular docking approach was employed with Mpro structures prepared for docking study. To do so, experimentally solved crystal structure of SARS-CoV-2 Mpro (PDB Id 6M2Q), HCoV-NL63 Mpro (PDB Id 5GWY), and HCoV-229E Mpro (PDB Id 2ZU2) were retrieved from the RCSB PDB database (www.rcsb.org). The retrieved structures were prepared for docking in Discovery Studio Visualizer [44] by removing the hetero atoms, water molecules and any co-crystallized ligand groups followed by the addition of polar hydrogen atoms.

Unfortunately, the crystal structure for HCoV-OC43 Mpro was not available in the PDB database and therefore, we performed the homology-based 3D structure modeling in the MODELLER 10.1 tool [40]. Briefly, the HCoV-OC43 Mpro protein sequence was submitted for structure-based similarity search in NCBI- BLAST (https://blast.ncbi.nlm.nih.gov/Blast.cgi) using RCSB PDB as the search database to obtain the best template for modeling and HCoV-HKU1 Mpro (PDB ID: 3D23) displayed a percent identity of 82.67% with the sequence of HCoV-OC43 Mpro as well as 99% query cover for structure modeling. A total of 50 models were prepared in Modeler 10.1 and were ranked based on their DOPE score. After loop refinement of the top model, the stereochemical analysis of the final structure was performed in SAVES v6.0 (PROCHECK) (https://saves.mbi.ucla.edu) [41] which showed the Ramachandran value of 95.5% amino acids in the most favored region while no residues in the disallowed region (Supplementary Figure 1). The overall quality assessment of the model structure was obtained from ProtSAV (www.scfbio-iitd.res.in/software/proteomics/protsav.jsp) [42]. The ProtSAV combines multiple stereochemical analysis parameters to plot and compute the global quality factor of the structure and results in the range of root mean square deviation (rmsd) value. The interpretation of the overall quality assessment suggests that the model structure is within the range of 2-3Å rmsd (Supplementary Figure 1). Lastly, the rmsd was also computed between the template (HCoV-HKU1 Mpro) and the HCOV-OC43 modeled structure by superimposing in Chimera tool [43] and we obtained a RMSD value of 0.171Å only (Supplementary Figure 1).

For virtual screening of small molecules, an FDA-approved drug library with 1576 compounds was retrieved from the ZINC15 database. Further, the compounds were prepared for docking studies in AutoDock Vina [46] and SwissDock [47]. The OpenBabel tool was used for compound preparation [45]. This tool allows the interconversion of chemical structures in many formats as required by particular docking tools. The compounds from ZINC15 database was obtained in 3D sdf format and compressed into a single file. For the AutoDock tool, the compounds were prepared in autodock pdbqt format by conversion from sdf format in the OpenBabel tool. The converted files were separated into individual compounds each with 3D coordinates with non-polar hydrogens merged and added with polar hydrogens and gasteiger charge through OpenBabel tool. Similarly for SwissDock also, the required mol2 files were interconverted from sdf format into the OpenBabel tool with all hydrogens and 3D coordinates.

**Figure 3.**
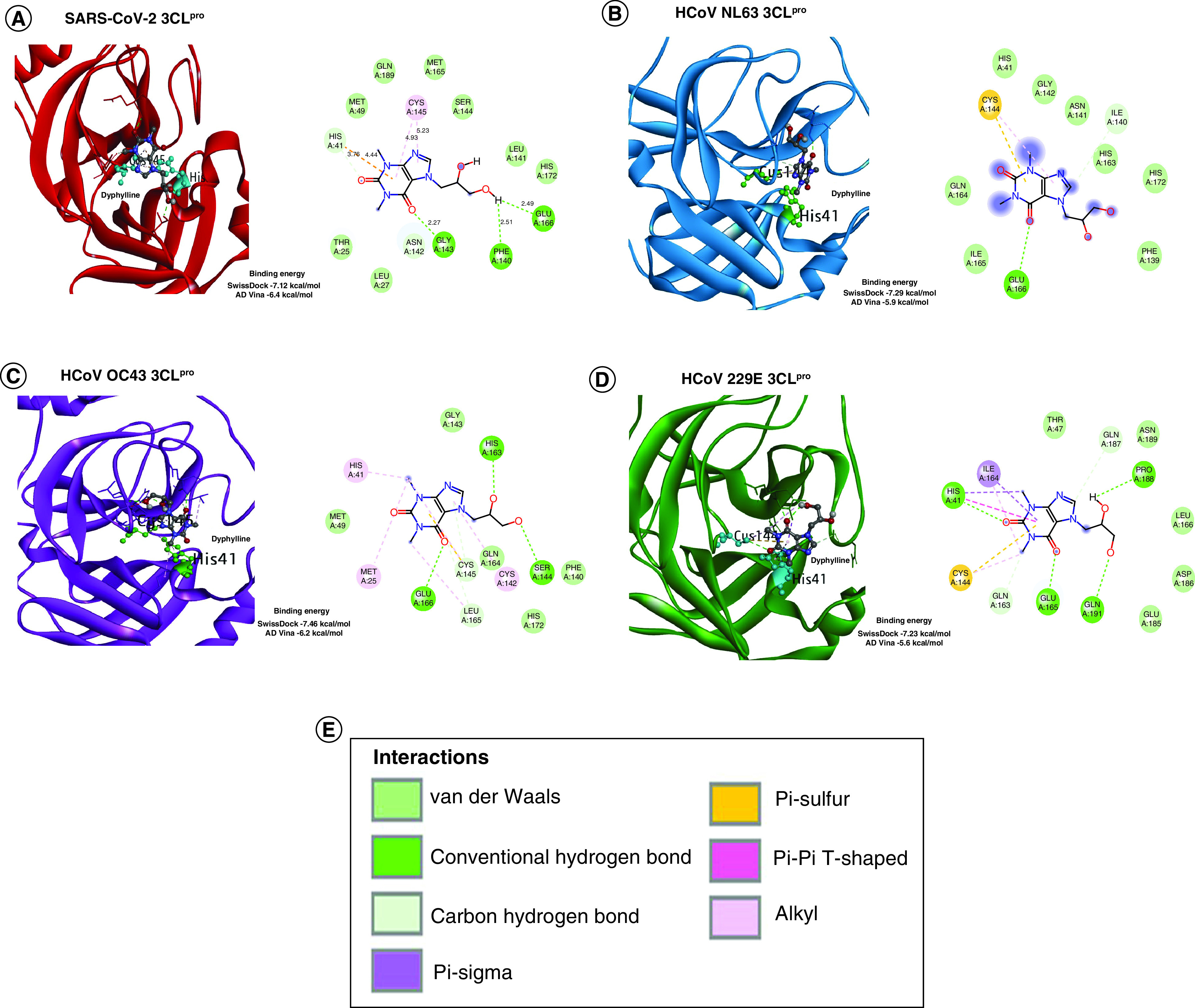
Molecular docking of dyphylline with Mpro/3CLpro of four Coronavirus strains. **(A)** SARS-CoV-2. **(B)** HCoV-NL63. **(C)** HCoV-OC43. **(D)** HCoV-229E. The blind docking was performed in AutoDock (AD) Vina and SwissDock and the protein-drug interaction was analyzed through Discovery studio visualizer. The binding affinity between drug and protein calculated from both docking tools in kcal/mol are provided and their different inter-molecular interaction type is coded with a specific color.

In both docking tools, the above prepared Mpro structures were sequentially docked with all the prepared compounds. An unbiased blind docking was performed to allow compounds to find the best fit solution with the target proteins. After docking, only those compounds whose binding energy score was ≤-5 kcal/mol were analyzed to check binding specificity for Mpro of each strain. The compound ZINC Id- ZINC57147, commonly known as dyphylline was the most apposite compound, interacting with the catalytic dyad of each Mpro structures in both docking results. Dyphylline made an intermolecular interaction with the catalytic dyad residue's histidine (H) and cystine (C) in each of the Mpro as well as displayed comparable binding energy with all four Mpro structures (Figure 3). We performed these intermolecular interaction analysis of each complex in discovery studio visualizer through protein-ligand interaction analysis section to find out the type of interactions between the catalytic dyad residues and dyphylline. In the case of SARS-CoV-2 Mpro and dyphylline complex, a total of 14 residues interacted with it and H41 was in hydrogen bond (HB) and pi-interaction while C145 was in alkyl-interactions (Figure 3A). The docking scores were −7.12 (SwissDock) and −6.4 (AutoDock Vina) kcal/mol, respectively. In HCoV-NL63 Mpro and dyphylline complex, a total of 11 residues interacted and H41 was in van der Waals (VdW) while C144 in alkyl and pi-interactions (Figure 3B). The docking scores were -7.29 (SwissDock) and −5.9 (AutoDock Vina) kcal/mol, respectively. Further, in HCoV-OC43 Mpro and dyphylline complex, a total of 13 residues interacted with it in which H41 was in alkyl-interaction while C145 was making HB (Figure 3C). The docking scores were −7.46 (SwissDock) and −6.2 (AutoDock Vina) kcal/mol, respectively. Finally, the interaction analysis between the complex of HCoV-229E Mpro and dyphylline revealed that out of a total 13 residues interactions, H41 was both in HB and pi-interactions while C144 was in alkyl and pi-interactions. The dock scores were −7.23 (SwissDock) and −5.6 (AutoDock Vina) kcal/mol, respectively (Figure 3D).

Overall, dyphylline displayed interactions with the catalytic dyad as its best fit docking site with Mpro of all four coronavirus strains and the cumulative energetic effect of non-covalent interactions between dyphylline and Mpro of SARS-CoV-2, HCoV-NL63, HCoV-OC43 and HCoV-229E are comparable. These results encouraged us to further validate the antiviral activity in cell culture models infected with coronaviruses.

### Antiviral effects of dyphylline in different cell culture models of HCoV-NL63

**Figure 4.**
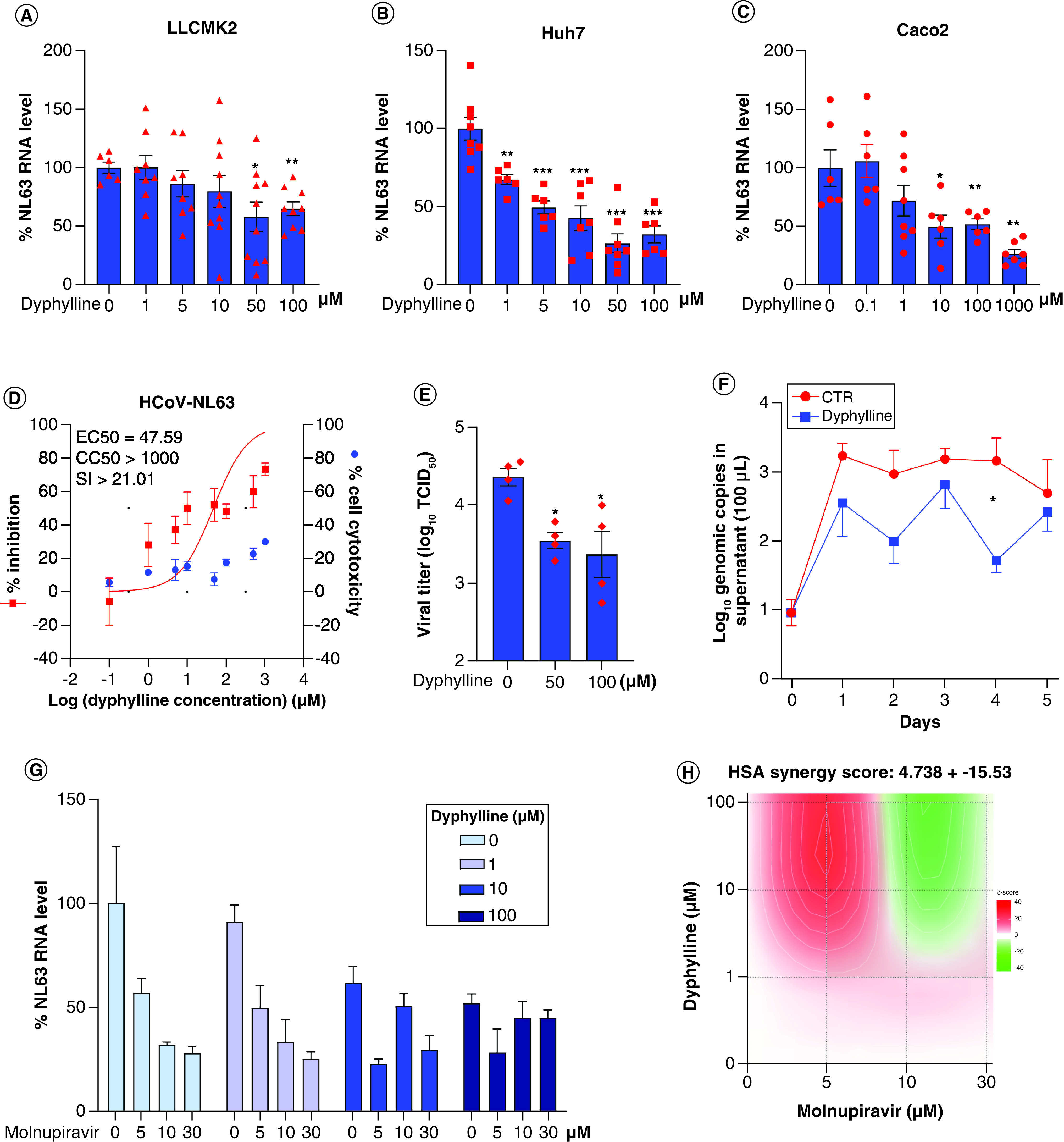
Antiviral effects of dyphylline against HCoV-NL63 in cell culture models. **(A–C)** The effects of dyphylline treatment on HCoV-NL63 replication in LLCMK-2, Huh7 and Caco-2 cell line models. Intracellular HCoV-NL63 RNA levels were normalized to reference gene GAPDH and the values were presented in relative to the control (CTR) (set as 1) (n = 6). **(D)** HCoV-NL63-infected Caco-2 cells were treated with different concentrations of dyphylline for 48 h. EC50 and CC50 curves were presented. The left and right Y-axis of the graphs indicates the mean % inhibition of viral RNA and cytotoxicity by the treatment, respectively (n = 6–16). **(E)** HCoV-NL63-infected Caco-2 cells were untreated or treated with 50 or 100 μM dyphylline for 48 h. Infectious virus titers were measured by TCID50 assay (n = 4). **(F)** HCoV-NL63 infected Caco-2 cells were untreated or treated with 50 μM dyphylline for 5 days. The supernatant was collected for quantifying secreted viruses by qRT-PCR, and calculated as genomic copy numbers (n = 6). The standard curve for calculating genomic copy numbers is included in Supplementary Figure 4. **(G)** The antiviral effects of combining various concentrations of dyphylline in combination with molnupiravir. **(H)** Synergy plot representing the score for the combination of dyphylline and molnupiravir based on the results shown in G (n = 4–6). Data represented as mean ± SEM. *p < 0.05; **p < 0.01; ***p < 0.001.

HCoV-NL63 is a seasonal coronavirus but has the same cell entry receptor as SARS-CoV-2. We inoculated LLCMK-2, Huh7 and Caco-2 cells with HCoV-NL63. Treatment of dyphylline dose-dependently inhibited viral RNA in all three cell models (Figure 4A–C), and minimal cytotoxicity was observed by dyphylline treatment (Supplementary Figure 2A–C). The half-maximum effective concentration (EC50) of dyphylline was 47.59 μM, and the half-maximum cytotoxic concentration (CC50) was above 1000 μM, resulting in a selectivity index (SI, CC50/EC50) higher than 20 (Figure 4D). A TCID50 assay was performed to measure the titer of secreted infectious HCoV-NL63 viruses in supernatant of Caco-2 cells harvested at 48 h after treatment of dyphylline. The titers of produced infectious particles were significantly reduced by dyphylline (Figure 4E). For example, incubation with 50 μM dyphylline resulted in a 81.64% ± 6.17 (mean ± SEM, n = 4, p < 0.0001) reduction of viral titer. Next, we further profiled the dynamics on the production of viral genomic RNA in a consecutive 5-day course by treatment with 50 μM dyphylline in Caco-2 cells. The secretion of viral RNA into supernatant was significantly inhibited by dyphylline (Figure 4F). For example, after treatment with dyphylline for 4 days, the level of secreted viral RNA was reduced by 94.64% ± 1.76 (mean ± SEM, n = 6; p < 0.0001).

A combination of antiviral drugs with different mechanism-of-actions is often used in the clinic to enhance antiviral activity and to prevent drug resistance development. We next tested the combination of dyphylline with molnupiravir, a recently authorized ribonucleoside analog for treating SARS-CoV-2 infection. In Caco-2 cells infected with HCoV-NL63, we observed a moderate additive effect with the HSA synergy score of 4.738 ± 15.53 (Figure 4G & H), and the drug concentrations had no apparent cytotoxicity on Caco2 cells (Supplementary Figure 2F).

### Pan-coronavirus antiviral effects of dyphylline in different coronavirus cell culture models

**Figure 5.**
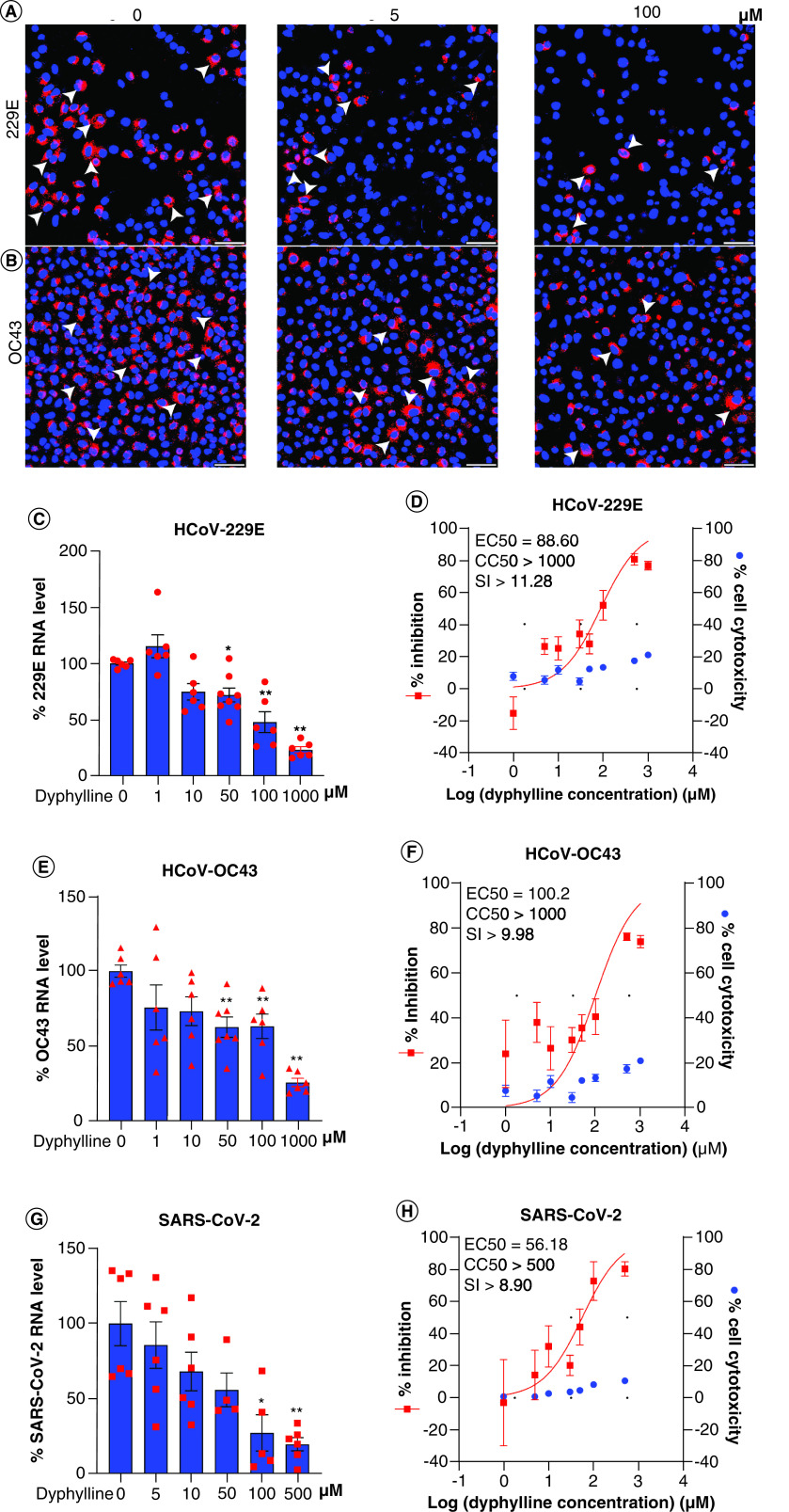
Antiviral activity of dyphylline against pan-coronavirus in cell culture models. **(A & B)** Immunofluorescence staining of dsRNA, the replicating intermediate of HCoV-229E or HCoV-OC43 genomic RNA, after treatment of dyphylline in A549 cells. Nuclei were stained by DAPI (blue). **(C, E & G)** The effects on HCoV-229E, HCoV-OC43 and SARS-CoV-2 replication in A549 or Calu-3 cell lines by dyphylline. Intracellular viral RNA levels were normalized to reference gene GAPDH and presented in relative to the control (CTR) (set as 1) (n = 4–8). **(D, F & H)** A549 or Calu-3 cells were inoculated with 0.1 MOI HCoV-229E, HCoV-OC43 or SARS-CoV-2, and treated with dyphylline for 48 h. EC50 and CC50 curves were presented. The left and right Y-axis of the graphs indicate the mean % inhibition of virus RNA and cytotoxicity of the treatment, respectively (n = 4–16). Data represented as mean ± SEM. *p < 0.05; **p < 0.01; ***p < 0.001.

To evaluate whether dyphylline has pan-coronavirus antiviral activity, we employed models of human A549 lung cell line infected with seasonal HCoV-229E and HCoV-OC43, and Calu3 lung cell line infected with SARS-CoV-2. Immunofluorescent staining of viral dsRNA showed a reduction of the number of infected cells by dyphylline treatment in 229E and OC43 (Figure 5A & B). This dose-dependent inhibitory effect was further confirmed by qRT-PCR quantification of cellular viral RNA in cell model, with minimal effects on cell viability (Figure 5C & E & Supplementary Figure 2D). The EC50 of dyphylline against HCoV-229E and HCoV-OC43 replication was 88.60 μM and 100.2 μM, respectively, and the CC50 was over 1000 μM, SI in HCoV-229E and HCoV-OC43 were both over 10 (Figure 5D & F). Similar to the findings on seasonal coronaviruses, dyphylline also has an antiviral effect in the Calu-3 cell line infected with SARS-CoV-2 (Figure 5G). The EC50 of dyphylline against SARS-CoV-2 replication was 56.18 μM, and CC50 was over 500 μM, SI was nearly over 10, which is similar to seasonal coronaviruses (Figure 5H).

## Discussion

Our study showed, through computational screening, that dyphylline can potentially block the catalytic dyad of Mpro and exhibited antiviral function in cell culture models of four HCoVs, including SARS-CoV-2.

HCoVs have been found to cause mild to severe respiratory diseases including ‘common cold’, pneumonia, bronchiolitis, hypoxia and life-threatening acute respiratory distress syndrome leading to death in many cases. The zoonotic coronaviruses from alpha and beta genera have emerged to be highly pathogenic to humans [1,3,6]. These include SARS-CoV-2, the cause of the COVID-19 pandemic, SARS-CoV, HCoV-NL63, HCoV-229E, HCoV-OC43, HCoV-HKU1 and MERS-CoV [5,6]. Pan-antiviral drugs for HCoVs are lacking. Several therapeutic molecules used in the past for the treatment SARS-CoV and other viral strains have been repurposed for the treatment of closely related SARS-CoV-2 strain [45,46]. There is an unmet need for novel antiviral drugs, which are effective against SARS-CoV-2 and other major seasonal HCoVs. The molecular docking tools provide an efficient way to expedite the virtual identification of such potential therapeutic molecules which can be tested experimentally and subsequently in clinical trials.

**Figure 6.**
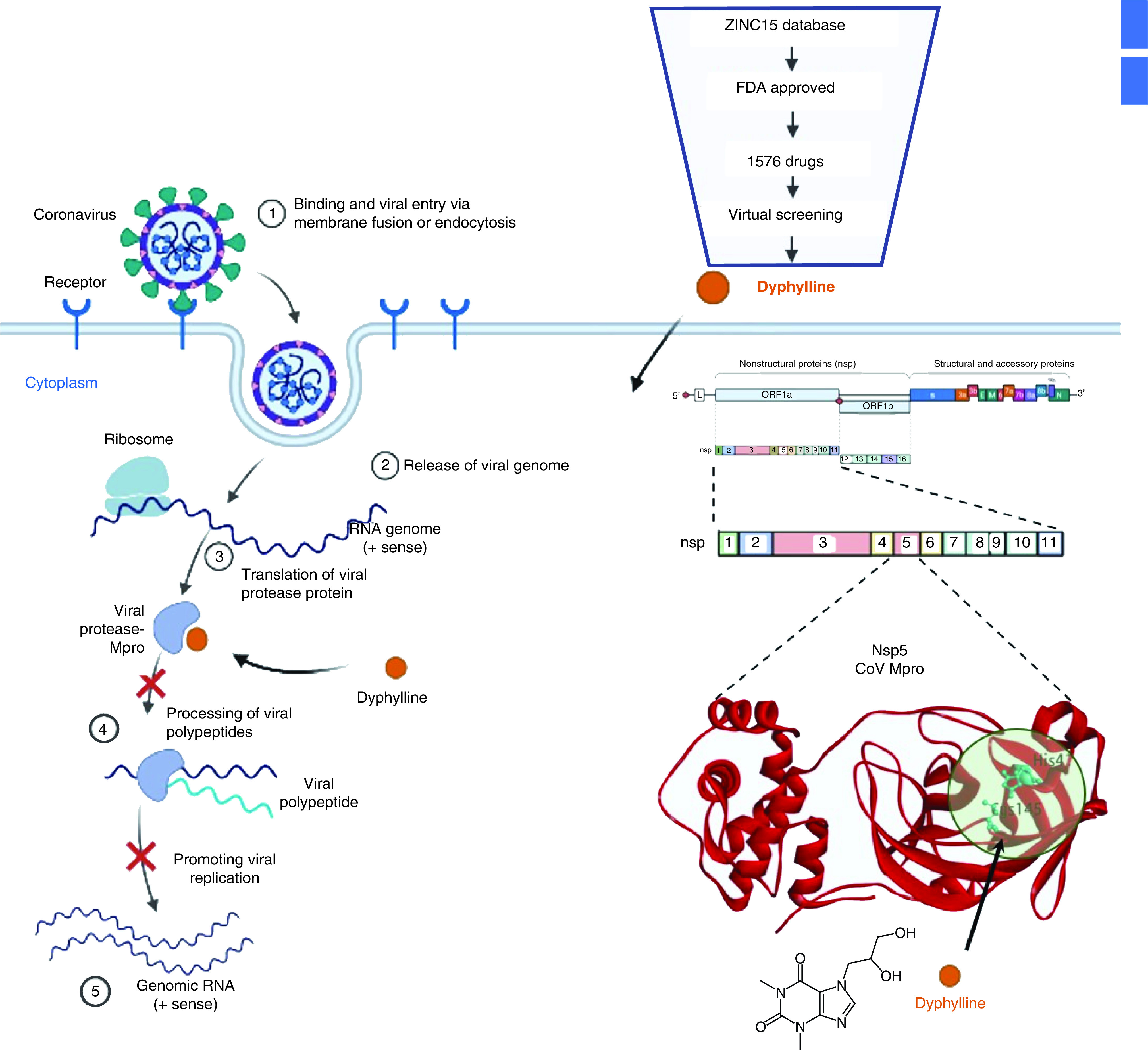
Computational approach to virtually screen dyphylline with inhibition potential to block the function of Mpro.

SARS-CoV-2 has multiple structural (spike, nucleocapsid) and non-structural (RdRp, Mpro, PLpro) proteins that are potential therapeutic targets [17,47,48]. HCoVs encode conserved protease known as Mpro or 3CLpro, which is mainly responsible for processing viral polypeptides into functional proteins and hence control viral translation and replication in the host system. This serves as a promising drug target to combat HCoVs infection and to alleviate the disease burden. Therefore, we planned to perform the study to screen and validate a novel molecule with an inhibition potential to block the function of Mpro as depicted in Figure 6.

Our study identified dyphylline that can block the catalytic dyad of Mpro from tested HCoV strains (SARS-CoV-2, HCOV-NL63, HCoV-229E, and HCoV-OC43) by virtual screening. Importantly, the dyphylline dose-dependently inhibited the tested four coronaviruses in cell culture models at variable concentrations ranging from 1 to 1000 μM. The EC50 and CC50 values indicate a therapeutic efficacy and safety against virus and cell lines, respectively. The EC50 value for HCoV-NL63, HCoV-229E, HCoV-OC43 and SARS-CoV-2 was obtained as 47.59, 88.60, 100.2, and 56.8 μM, respectively. On the other hand, the CC50 value for dyphylline was obtained as over 500 μM in SARS-CoV-2 infected Calu-3 cells while over 1000 μM for different cell lines infected with the other three strains. Interestingly, the antiviral effects of dyphylline were additive with molnupiravir, a recently authorized ribonucleoside analog that inhibits the replication of SARS-CoV-2 [11,49].

Recently, many investigators, including us, have studied the therapeutic potential of plant-based natural products including Xanthine derivatives, against the Mpro and other targets of SARS-CoV-2 and highly coveted its role in treatment of COVID-19 [17,50–53]. Xanthine derivatives have been shown to have the anti-inflammatory and immunomodulatory properties and are used in the treatment of respiratory diseases. Previous studies report the potential inhibitory effect of such xanthine derivatives, mainly pentoxifylline and caffeine-containing compounds against the Mpro of SARS-CoV-2 [29,54–56]. Dyphylline is well tolerated and has known drug efficacy as a potent bronchodilator to manage asthma and bronchitis [57]. Dyphylline may act synergistically to provide two clinical benefits: alleviating symptoms of bronchospasm and as an antiviral. Collectively, our molecular docking and *in vitro* experimental findings demonstrated the pan-coronavirus antiviral activity of dyphylline and support potential repurposing for treating coronavirus-infected patients. However, further validation in animal models is warranted before clinical applications.

**Summary points**

- Human coronaviruses (HCoVs) have evolved to be highly replicating pathogenic viruses. SARS-CoV-2 responsible for the COVID-19 pandemic is one of the most lethal outbreaks of HCoV and there is a pressing need for pan-HCoV therapies.
- The Mpro is largely conserved among HCoV and is responsible for processing the non-structural polypeptides into functional proteins and hence considered a potential therapeutic target.
- We therapeutically target the Mpro of HCoV-NL63 and HCoV-229E (from alpha-CoV genus) and HCoV-OC43 and SARS-CoV-2 (from beta-CoV genus).
- Through virtual screening, dyphylline, a xanthine derivative, was identified as an approved drug that potentially binds the catalytic dyad residue's histidine and cystine of all four Mpro structures *in silico*.
- In *in vitro* cell culture systems, dyphylline blocked viral replication of four tested HCoVs including SARS-CoV-2 in dose-dependent manner making this a candidate drug for pan-HCoV therapy.
