# Supplementary material for: Repurposing dyphylline as a pan-coronavirus antiviral therapy
Source: Future Med Chem. 2022 Apr 7;14(10):685–99. doi: 10.4155/fmc-2021-0311 (PMC9048854; doi:10.4155/fmc-2021-0311)
Supplement: Supplementary file 1 [file fmc-14-685-s1.docx]

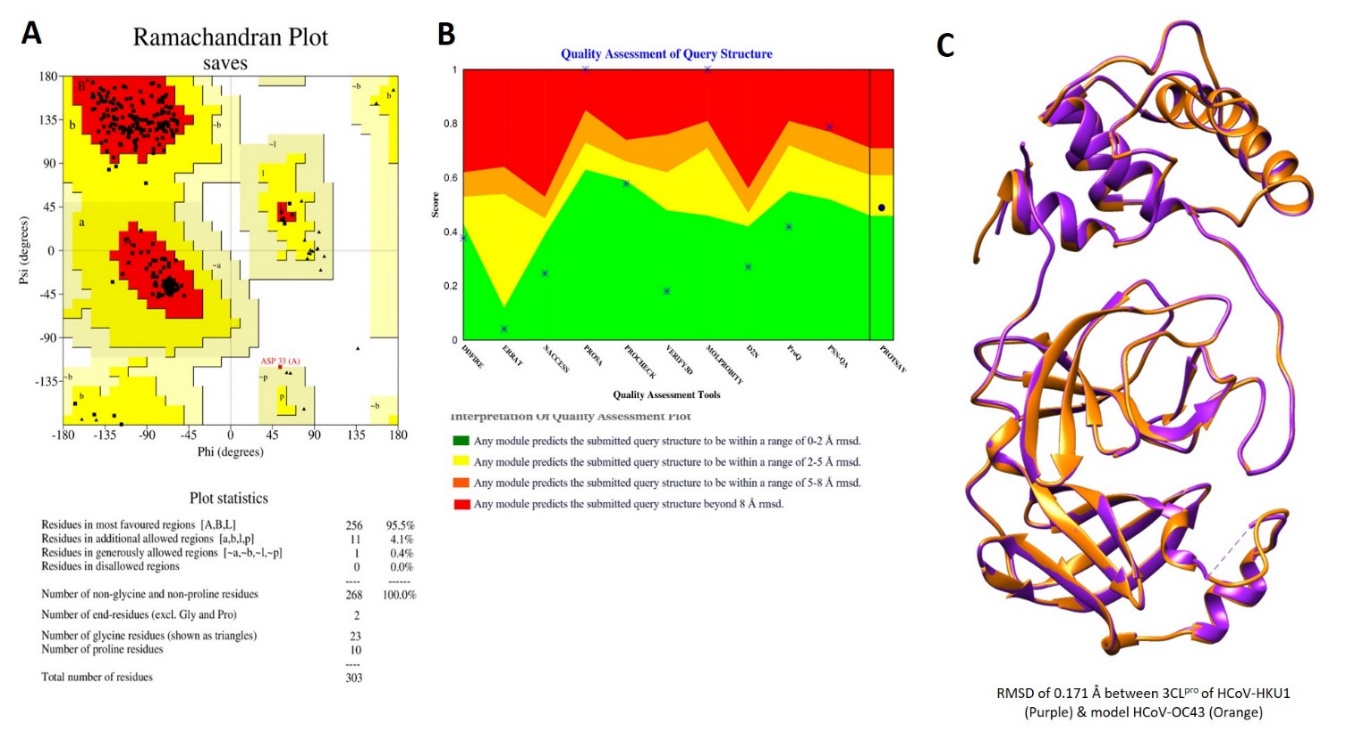
**Supplementary Files**

**Supplementary Figure S1**: Homology based 3D structure modelling of HCoV-OC43 main protease (Mpro)/3-Chymotrypsin-like protease (3CLpro). The HCoV-HKU1 Mpro structure (with 82.33% sequence identity and 99% query cover) was used as template and a total of 20 structures were modelled in the Modeller 10.1 tool and the HCoV-OC43 structure with lowest DOPE score was selected for further analysis and docking study. (A) The stereochemical analysis of the modelled HCoV-OC43 by the Ramachandran Plot produced a score of 95.5% residues in most favoured region while no residue in disallowed region, (B) Overall global quality assessment from multiple stereochemical parameters of structure on ProtSAV tool, and (B) Superimposition of template (HCoV-HKU1 Mpro) and model structure (HCoV-OC43 Mpro) in Chimera tool results only in 0.171Å root mean square deviation (RMSD) value.

**Supplementary Table 1. Primers used in the study**

| Gene name | F-sequence (5' to 3') | R-sequence (5' to 3') |
| --- | --- | --- |
| HCoV-NL63 plasmid | CTTCTGGTGACGCTAGTACAGCTTAT | AGACGTCGTTGTAGATCCCTAACAT |
| Human GAPDH | GTCTCCTCTGACTTCAACAGCG | ACCACCCTGTTGCTGTAGTAGCCA A |
| NL63 | ACGCAATGCCACTGTTGTTA | GACAACACCGTCATCAGAGA |
| 229E | GTCGTCAGGGTAGAATACCTTA | CCCGTTTGCGCTTTCTAGT |
| OC43 | AGCAACCAGGCTGATGTCAATACC | AGCAGACCTTCCTGAGCCTTCAAT |
| SARS-CoV-2 | CAATGGTTTAACAGGCACAGG | CTCAAGTGTCTGTGGATCACG |


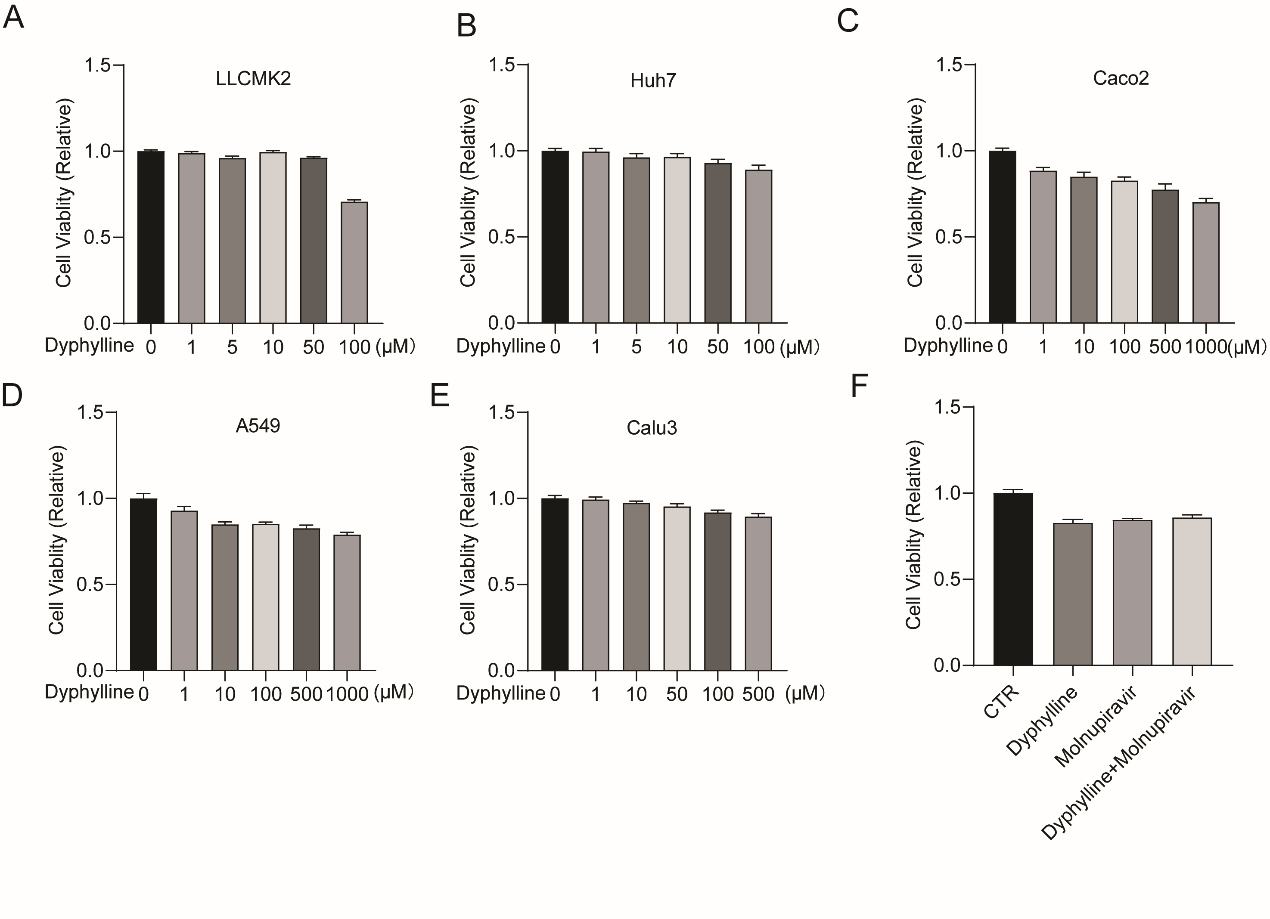


**Supplementary Figure S2**. The cytotoxicity of dyphylline on different cell lines. (A), (B), (C), (D) and (E) LLCMK-2, Huh7, Caco-2, A549 and Calu-3 cells treated with different concentrations of dyphylline for 48 h. Cytotoxicity was determined by MTT assay (n = 10-16). (F) Cytotoxicity of dyphylline, molnupiravir or their combinations. Caco-2 cells were treated with different concentrations of dyphylline (100 µM), molnupiravir (30 µM) or their combinations respectively for 48 h. Cytotoxicity was determined by MTT assay (n = 13-19).


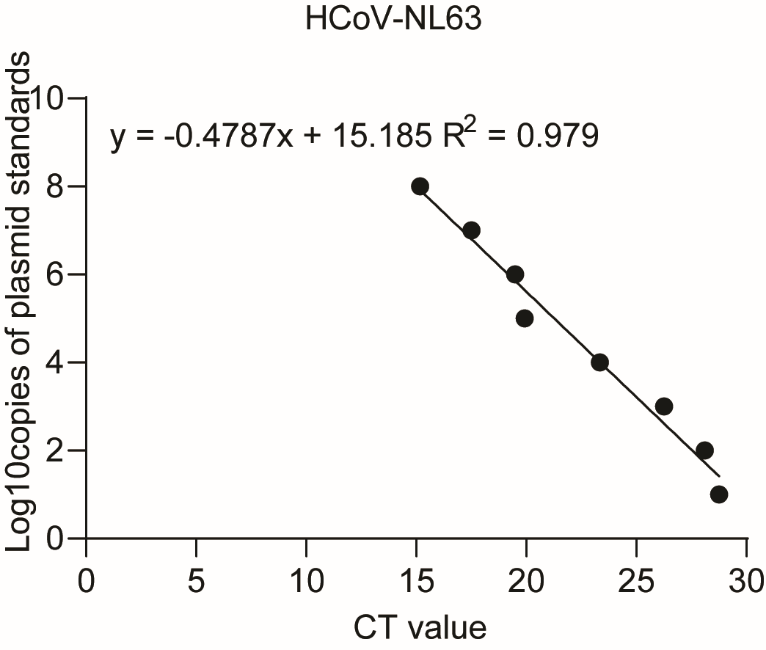


**Supplementary Figure S3.** Standard curve for quantifying HCoV-NL63 genome copy numbers. Amplicon of the N protein of HCoV-NL63 was cloned into the pCR2.1-TOPO vector. The plasmid was extracted, followed by a series of dilutions from 10^-1^ to 10^-8^ and then were amplified and quantified by qRT-PCR. Standard curve was generated by plotting the cycle threshold (CT) value regarding the log copy numbers.
